# A Rare Coexistence of Anti-CN1A and Anti-NXP2 Myositis-Specific Antibodies in a 63-Year-Old Female From the Philippines With Chronic Progressive Myopathy: A Case Report

**DOI:** 10.7759/cureus.106082

**Published:** 2026-03-29

**Authors:** Pamela Marie M Cope, Jerwin C Evangelista, Marianne B Libiran, Ludwig F Damian

**Affiliations:** 1 Institute for Neurosciences, St. Luke's Medical Center, Quezon City, PHL; 2 Neurology, St. Luke's Medical Center, Quezon City, PHL

**Keywords:** anti-cn1a, anti-nxp2, autoimmune, dermatomyositis, myositis

## Abstract

It is rare for two myositis-specific antibodies to co-exist in patients with immune-inflammatory myopathy. This overlap challenges the assumption of mutual exclusivity and may lead to conflicting diagnostic and therapeutic considerations. In this case, a 63-year-old female from the Philippines initially presented with chronic, progressive, and symmetrical proximal muscle weakness without cutaneous manifestations. Electrodiagnostic evaluation revealed a myopathic process while total creatine kinase levels were only mildly elevated. The myositis-specific antibody panel identified the presence of two distinct antibodies: anti-CN1A, which is a biomarker for inclusion body myositis, and anti-NXP2, which is specific to dermatomyositis. A muscle biopsy was performed, revealing fascicular atrophy, a characteristic feature of dermatomyositis. These findings presented a unique diagnostic challenge as dermatomyositis typically responds to immunosuppressive therapies, while inclusion body myositis is resistant to such treatments. This case illustrates the diagnostic complexity of overlapping myositis-specific antibodies and emphasizes the importance of integrating clinical, serological, and histopathological findings.

## Introduction

Dermatomyositis (DM) is the representative example of inflammatory myopathies. It most commonly affects individuals between 30 and 60 years of age, and a smaller group shows a peak incidence at 15 years of age. Women are more frequently affected across all age groups [1].

Individuals with this condition typically present with a gradual, insidious onset of painless proximal muscle weakness that progressively worsens over several weeks to months. Commonly, they would initially complain of difficulty getting up from a chair, climbing stairs, walking, and lifting their arms above their head [1]. In addition to muscle involvement, it is crucial to search for signs and symptoms of other systemic involvement, particularly skin lesions characteristic of dermatomyositis. These include periorbital blue-purple rash (heliotrope rash), erythematous rash on the face, anterior chest (V sign), shoulders (shawl sign), and lateral hips (holster sign), and a violaceous eruption on the knuckles (Gottron papules) [2].

Laboratory and diagnostic studies support the diagnosis of dermatomyositis. Creatine kinase (CK) levels in individuals with dermatomyositis may be markedly elevated, although in some cases they can be normal or only mildly elevated. Electromyography is often performed in dermatomyositis to rule out other causes of weakness, typically revealing myopathic characteristics like short-duration, small-amplitude motor unit action potentials [2]. Muscle biopsy in dermatomyositis can be helpful for diagnosis when perifascicular atrophy is present, although this hallmark feature is seen in only about 50% of cases and is often not required for diagnosis if typical skin manifestations or specific autoantibodies are present [2].

There are several antibodies specific to dermatomyositis, and these antibodies are linked to specific clinical presentations.

Anti-Mi-2 antibodies are present in 2-38% of adult dermatomyositis and are characterized by classic dermatomyositis with prominent cutaneous manifestations. They often have an exceptional weakness and have the highest CK levels, but have a favorable prognosis. Anti-NXP2 antibodies are associated with substantial muscle weakness, including distal weakness, dysphagia, and a notable association with calcinosis [2]. These individuals have an increased risk for malignancy within three years of diagnosis. Anti-TIFI1γ antibodies are most strongly associated with underlying malignancy, with a tumor rate of approximately 20%-65%. Anti-MDA5, on the other hand, is prevalent in most adults with clinically amyopathic dermatomyositis. This subtype is linked to an increased risk of rapidly progressive interstitial lung disease (ILD), often refractory to immunosuppression, and carries a high mortality rate. Lastly, anti-SAE antibodies manifest with profound cutaneous signs and minimal initial myopathy [2].

Inclusion body myositis (IBM) is another classification of immune-inflammatory myopathy and affects individuals older than 50 years, characterized by steadily progressive, painless muscular weakness and modest atrophy, often presenting with focal or asymmetric weakness in the quadriceps, finger/wrist flexors, or lower leg muscles [3]. It is uniquely associated with the anti-CN1A antibody in two-thirds of patients, which is specific to IBM and aids in differentiating it from other inflammatory myopathies.

Despite advances in the characterization of myositis-specific antibodies, the coexistence of multiple antibodies remains uncommon and is not well understood. This overlap challenges the traditional assumption of mutual exclusivity and may lead to diagnostic uncertainty and therapeutic dilemmas.

This article was previously presented as a poster at the 2025 World Congress of Neurology on October 12-15, 2025, in Seoul, Korea.

## Case presentation

We present a case of a 63-year-old female from the Philippines who presented in October 2023 with a nine-month history of chronic, progressive, and symmetric, non-fatigable proximal weakness. There was no diplopia, dysphagia, respiratory symptoms, joint pains, sensory complaints, antecedent infection, or vaccination prior to symptom onset. Notably, there were no initial cutaneous manifestations at presentation. On neurological examination, there was notable proximal muscle atrophy in both upper and lower extremities. No fasciculations, percussion myotonia, tremors, or contractures were observed. Manual muscle testing of the upper extremities revealed a symmetric 4/5 strength across all major myotomes, specifically involving shoulder abduction and adduction (C5), elbow flexion (C6), elbow extension (C7), and wrist and finger flexion (C8). Additionally, both neck flexors and extensors demonstrated a reduced strength of 4/5. In the lower extremities, hip flexion, extension, abduction, and adduction (L2-L5) were graded at 3/5 bilaterally. Strength of knee flexion and extension (L3-S1), ankle dorsiflexion (L4-L5), great toe extension (L5), and plantarflexion (S1-S2) were graded 4/5 bilaterally.

Deep tendon reflexes were symmetric but diminished throughout, specifically graded at 1+ for the bilateral biceps, brachioradialis, triceps, patellar, and ankle.

**Figure 1:**
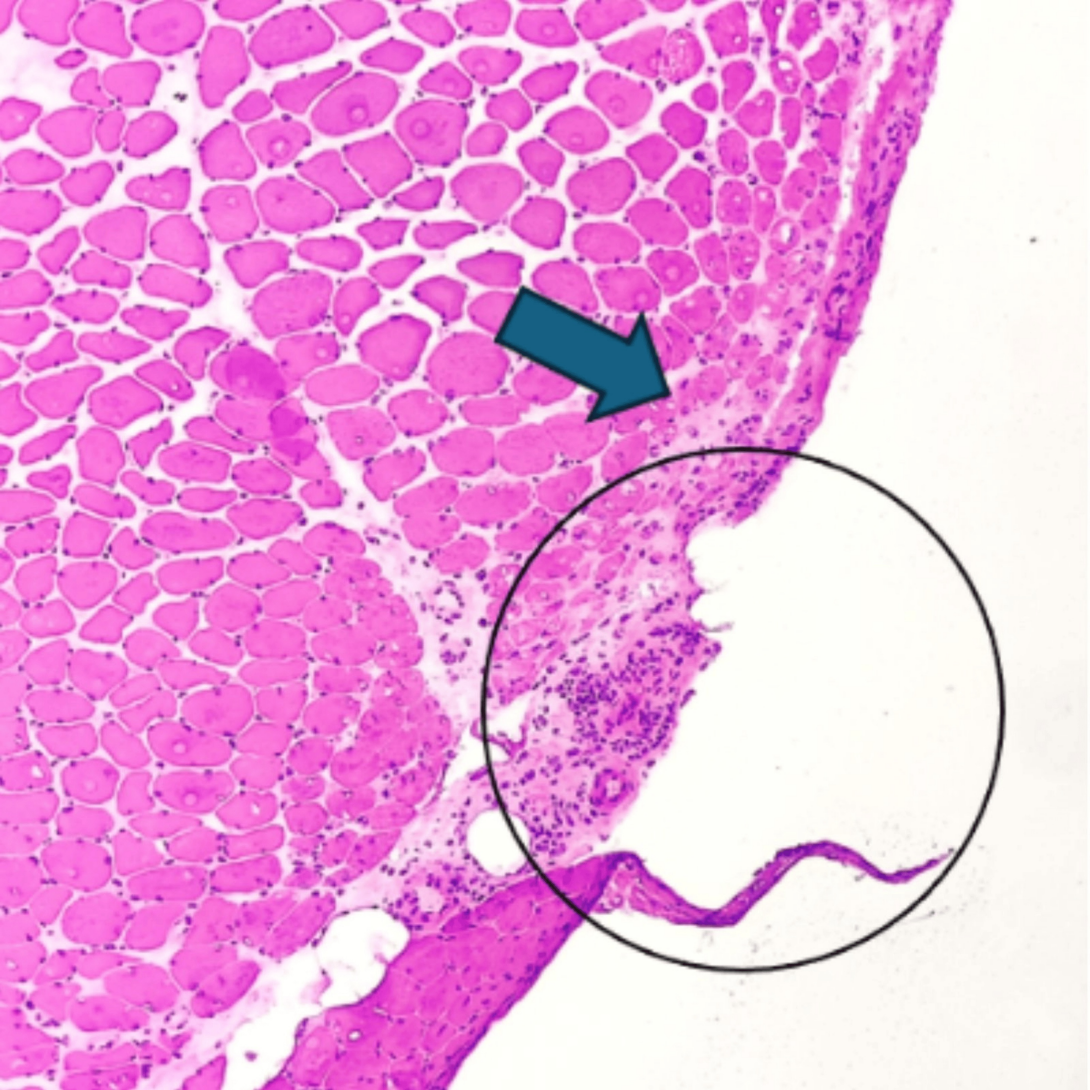
H&E-stained photomicrograph of transverse muscle section demonstrating predominantly normal-sized muscle fibers with perifascicular atrophy (arrow).

As shown in Table 1, laboratory and diagnostic findings revealed only a mildly elevated CK level of 251 u/L. Electromyography (EMG) and nerve conduction velocity (NCV) demonstrated normal sensory nerve conduction studies and highly polyphasic potentials and an early recruitment pattern, which are consistent with myopathies. Anti-nuclear antibody (ANA) was positive at 1:320, prompting anti-double-stranded DNA (anti-dsDNA) testing to rule out systemic lupus erythematosus (SLE) as a possible primary condition or overlap with certain forms of myositis; however, the anti-dsDNA result was negative. A subsequent myositis antibody panel revealed positivity for two antibodies: NXP2, commonly associated with dermatomyositis, and anti-CN1A, a marker for IBM. This created a diagnostic dilemma, as both dermatomyositis and IBM are immune-mediated inflammatory myopathies, but they are distinct entities with different therapeutic approaches. A muscle biopsy was later performed, showing perifascicular atrophy, the hallmark finding of dermatomyositis, as shown in Figure 1.

**Table 1:** Key laboratory findings. CK: creatine kinase; ANA: anti-nuclear antibody; Anti-dsDNA: anti-double-stranded DNA; Anti-cN1A: anti-cytosolic 5'-nucleotidase 1A; Anti-NXP2: anti-nuclear matrix protein 2.

Test	Result	Reference range
CK	251 u/L	22-200 U/L
ANA	1:320	<1:80
Anti-dsDNA	Negative	<10-15 IU/mL
Myositis panel	(+) Anti-cN1A, (+) Anti-NXP2	

Given the association of dermatomyositis with malignancy, the patient underwent a positron emission tomography (PET) scan, which was unremarkable for underlying malignancy.

Based on the clinical presentation, myopathic EMG findings, positive anti-NXP2 antibody, and a muscle biopsy showing perifascicular atrophy, a diagnosis of dermatomyositis was established despite the concurrent presence of anti-CN1A antibody.

Three months after the diagnosis, the patient developed non-pruritic, reddish-purple patches over the forehead, nasal bridge, and maxillary regions. She received pulse steroid therapy consisting of intravenous methylprednisolone 250 mg administered every six hours (totaling 1 g/day) for three consecutive days. Following pulse therapy, the patient was transitioned to oral prednisone at 1 mg/kg/day (60 mg/day) and was slowly tapered over a three-month period. This led to a significant improvement in both cutaneous manifestations and motor function. During this time, manual muscle testing revealed that strength in all upper extremity myotomes, as well as the neck flexors and extensors, had improved to 5/5. In the lower extremities, strength across all myotomes improved to 4/5 bilaterally. This represented a notable recovery in the previously 3/5 graded proximal girdle, specifically involving hip flexion, extension, abduction, and adduction.

## Discussion

The case of a 63-year-old female from the Philippines presenting with chronic, progressive muscle weakness and the co-detection of anti-CN1A and anti-NXP2 myositis-specific antibodies represents a significant diagnostic challenge. Anti-NXP2 antibodies are specific to dermatomyositis, associated with substantial muscle weakness, dysphagia, calcinosis, and, in adults, an increased risk of malignancy within three years. Dermatomyositis is often histologically characterized by perifascicular atrophy, observed in about 50% of cases, and typically responds well to immunosuppressive therapies [2]. The patient's eventual development of characteristic reddish-purple patches and the muscle biopsy finding of perifascicular atrophy strongly supported a diagnosis of dermatomyositis.

In contrast, anti-CN1A antibodies are uniquely associated with IBM, which primarily affects individuals over 50 years old. IBM is characterized by steadily progressive, painless muscular weakness, often focal or asymmetric, and is notably resistant to immunosuppressive treatment. Anti-cN1A seropositivity has not been linked to an increased risk of malignancy [3].

The coexistence of multiple myositis-specific antibodies like anti-CN1A and anti-NXP2 in a single patient is rare, as these antibodies are traditionally considered mutually exclusive when detected by immunoprecipitation (IP) methods. However, commercial line blot (LB) immunoassays frequently report such instances. In large cohorts, the detection of multiple myositis-specific antibodies occurs in only 0.11% to 2.1% of patients when using IP-based methods, whereas commercial LB or dot immunoassays report much higher frequencies, ranging from 3.4% to 16.7%. This discrepancy is largely attributed to the specificity issues of multiplex immunoassays [4]. Studies indicate that line blot testing for myositis antibodies in patients with suspected idiopathic inflammatory myopathy has a modest positive predictive value of approximately 63%, which drops significantly to 47% for weak-positive results [5]. Despite this, individual antibodies detected by line blot often demonstrate high specificity, exceeding 97.5% [6].

Beyond laboratory discrepancies, the potential biological mechanism for "true" antibody coexistence must be considered. One leading hypothesis is "intermolecular epitope spreading," where ongoing target organ inflammation leads to the continuous exposure of various intracellular autoantigens to the immune system. This chronic exposure can broaden the immune response, causing the system to "spread" its targeting from one antigen to another over time. Additionally, shared genetic predispositions, such as the HLA-DRB1*03:01 allele, which is strongly associated with various myositis subtypes, may facilitate the development of multiple autoantibody specificities in a single individual [6].

Crucially, the patient's positive response to pulse steroid therapy, with significant improvement in muscle strength and skin rashes, aligns with the expected treatment response in dermatomyositis and sharply contrasts with IBM. This diagnostic trajectory serves as a vital clinicopathological correction. While the serological profile was ambiguous, the integration of histological hallmarks, specifically perifascicular atrophy and the functional therapeutic response, allowed for the correction of the diagnosis toward a clinically dominant dermatomyositis phenotype.

This case, therefore, emphasizes the critical importance of integrating clinical, serological, and histopathological findings for accurate diagnosis and effective management, particularly when facing overlapping or conflicting antibody profiles.

Further supporting the diagnostic consideration, anti-NXP2 antibodies in adult dermatomyositis have been consistently associated with a distinct clinical phenotype and an increased risk of malignancy. Studies have demonstrated that a significant proportion of patients with cancer-associated dermatomyositis harbor antibodies against nuclear matrix protein 2, reinforcing the importance of malignancy screening in this subgroup. Additionally, anti-NXP2 positivity has been linked to more severe muscle involvement and systemic manifestations, aligning with the patient’s progressive proximal weakness and cutaneous findings [7,8].

From a broader diagnostic framework, the classification of idiopathic inflammatory myopathies underscores the importance of integrating serological, clinical, and histopathological domains. The 2017 European League Against Rheumatism/American College of Rheumatology classification criteria emphasize that no single parameter is sufficient for diagnosis, particularly in cases with atypical or overlapping antibody profiles. In parallel, IBM remains primarily a clinicopathological diagnosis characterized by treatment-refractory progression, which contrasts with the observed steroid responsiveness in this case. These established principles further support the interpretation that the patient’s presentation is most consistent with a dermatomyositis phenotype despite conflicting serological findings [9,10].

Limitations of this report include the single-patient design and reliance on commercial line blot assays, which may yield false-positive results and limit generalizability.

## Conclusions

This report presents the rare coexistence of anti-CN1A and anti-NXP2 antibodies, highlighting the diagnostic and therapeutic challenges when multiple myositis-specific antibodies are detected. Despite the presence of an IBM-associated antibody, the patient exhibited a clinically dominant, steroid-responsive dermatomyositis phenotype. This underscores the importance of integrating serology with clinical and histopathological findings rather than relying on antibody results alone to guide management in complex inflammatory myopathies.

## References

[1] Ropper AH, Samuels MA, Klein JP, Prasad S (2023). Adams and Victor's Principles of Neurology. https://accessmedicine.mhmedical.com/book.aspx?bookid=3313.

[2] Jammal C, Pinal-Fernandez I, Mammen AL (2024). Adult inflammatory myopathies: updates on classification and management. Pract Neurol.

[3] Salam S, Dimachkie MM, Hanna MG, Machado PM (2022). Diagnostic and prognostic value of anti-cN1A antibodies in inclusion body myositis. Clin Exp Rheumatol.

[4] Van Horebeek N, Vulsteke JB, Bossuyt X (2021). Detection of multiple myositis-specific autoantibodies in unique patients with idiopathic inflammatory myopathy: a single centre-experience and literature review: systematic review. Semin Arthritis Rheum.

[5] Chang YC, Yang L, Budhram A (2024). Positive predictive value of myositis antibody line blot testing in patients with suspected idiopathic inflammatory myopathy. Muscle Nerve.

[6] Kleiser B, Hoffmann D, Kowarik MC, Dubois E, Armbruster M, Grimm A, Marquetand J (2024). Myositis-specific and -associated antibodies in neurological disorders - a retrospective study of 727 patients. J Neurol Sci.

[7] Fiorentino DF, Chung LS, Christopher-Stine L (2013). Most patients with cancer-associated dermatomyositis have antibodies to nuclear matrix protein NXP-2 or transcription intermediary factor 1γ. Arthritis Rheum.

[8] Rogers A, Chung L, Li S, Casciola-Rosen L, Fiorentino DF (2017). Cutaneous and systemic findings associated with nuclear matrix protein 2 antibodies in adult dermatomyositis patients. Arthritis Care Res (Hoboken).

[9] Greenberg SA (2019). Inclusion body myositis: clinical features and pathogenesis. Nat Rev Rheumatol.

[10] Lundberg IE, Tjärnlund A, Bottai M (2017). 2017 European League Against Rheumatism/American College of Rheumatology classification criteria for adult and juvenile idiopathic inflammatory myopathies and their major subgroups. Ann Rheum Dis.

